# Supplementary material for: Assessment of inter-rater and intra-rater reliability of the Luna EMG robot as a tool for assessing upper limb proprioception in patients with stroke—a prospective observational study
Source: PeerJ. 2024 Aug 29;12:e17903. doi: 10.7717/peerj.17903 (PMC11366229; doi:10.7717/peerj.17903)
Supplement: Supplemental Information 2 [file peerj-12-17903-s002.docx]

STROBE Statement—checklist of items that should be included in reports of observational studies

|  | Item No. | Recommendation | Page  No. | Relevant text from manuscript |
| --- | --- | --- | --- | --- |
| **Title and abstract** | 1 | (*a*) Indicate the study’s design with a commonly used term in the title or the abstract | 1 | Assessment of inter-rater and intra-rater reliability of the Luna EMG robot as a tool for assessing upper limb proprioception in patients with stroke - a prospective observational study |
|  |  | (*b*) Provide in the abstract an informative and balanced summary of what was done and what was found | 1 | Measurements performed by the Luna EMG diagnostic and rehabilitation robot demonstrate high inter-rater and intra-rater agreement in the assessment of upper limb proprioception in patients with chronic stroke. The findings show that Luna EMG is a reliable tool enabling effective evaluation of upper limb proprioception post-stroke. |
| Introduction | | | |  |
| Background/rationale | 2 | Explain the scientific background and rationale for the investigation being reported | 2-3 | Novel methods and techniques are mainly intended to enable patients with stroke to improve their functioning within the society. Today, functional therapy is frequently designed to apply a biofeedback method, with the use of robotic devices (Kołcz-Trzęsicka et al., 2017; Kwolek et al., 2013). One of such devices, applied in rehabilitation of the affected upper limbs, is LUNA EMG. In its operation it uses reactive electromyography to train the sensorimotor cortex (Olczak el al., 2021; Zasadzka et al., 2020).  In the literature there are few reports focusing on the use of the Luna EMG robotic device in assessment and rehabilitation of upper extremity (Clinical Trials: Efficacy 2023). One study investigated inter-rater and intra-rater reliability of Luna EMG in the assessment of upper limb proprioception, however it was conducted in a group of healthy individuals (Leszczak et al., 2024). |
| Objectives | 3 | State specific objectives, including any prespecified hypotheses | 3 | Therefore the current study was designed to examine inter-rater and intra-rater reliability of the Luna EMG multifunction robotic device as a tool intended to assess upper limb proprioception in patients with a stroke. |
| Methods | | | |  |
| Study design | 4 | Present key elements of study design early in the paper | 3 | The research was conducted in a form of a prospective observational study.  It was carried out in accordance with the ethical rules of the Declaration of Helsinki, and approved by the Local Bioethics Commission of the University of Rzeszow (resolution no. 2022/036/W). Written consent was obtained from all the participants. The study was registered in the clinical trials register at ClinicalTrials.gov (registration number NCT05486052). |
| Setting | 5 | Describe the setting, locations, and relevant dates, including periods of recruitment, exposure, follow-up, and data collection | 3 | The study was conducted in a rehabilitation and spa therapy hospital in the Podkarpackie Region, Poland |
| Participants | 6 | (*a*) *Cohort study*—Give the eligibility criteria, and the sources and methods of selection of participants. Describe methods of follow-up  *Case-control study*—Give the eligibility criteria, and the sources and methods of case ascertainment and control selection. Give the rationale for the choice of cases and controls  *Cross-sectional study*—Give the eligibility criteria, and the sources and methods of selection of participants | 3-4 | The inclusion criteria were defined as follows: completed first ischemic stroke; patient’s informed, voluntary consent to participate; elementary (basic) gripping ability; the upper limb and hand paresis rated 4-5 on the Brunnström scale; degree of disability Rankin score of 3; spastic tension of the upper limb and hand paresis not more than 3 on the modified Ashworth scale; and current health condition, confirmed by a medical examination, allowing participation in tests and exercises. The following exclusion criteria were applied: lack of the patient’s informed, voluntary consent; subsequent stroke; haemorrhagic stroke; stroke of the brainstem; cerebellum disorders of the higher mental functions limiting the ability to understand and perform tasks during examinations; visual disturbances; mechanical and thermal injuries potentially impairing the hand grip function; concomitant neurological, rheumatological and orthopaedic diseases, including permanent contractures that may affect the grasping ability and locomotion; unstable medical condition; metal implants; electronic implants; menstruation in women and epilepsy.  There were 200 patients with stroke in the rehabilitation and spa therapy hospital at the time of the study. Based on the inclusion and exclusion criteria, 147 patients were initially qualified to participate, however after medical examination eight individuals were excluded. Ultimately 130 patients took part in Examination I, and 126 patients participated in Examination II (Figure 1). |
|  |  | (*b*) *Cohort study*—For matched studies, give matching criteria and number of exposed and unexposed  *Case-control study*—For matched studies, give matching criteria and the number of controls per case |  |  |
| Variables | 7 | Clearly define all outcomes, exposures, predictors, potential confounders, and effect modifiers. Give diagnostic criteria, if applicable | 4 | The analysis concerned a homogeneous group. In addition, the study did not last long, so if there were any additional factors, they probably worked in the same way throughout the study period, so they should not affect the results of the repeatability analysis. |
| Data sources/ measurement | 8* | For each variable of interest, give sources of data and details of methods of assessment (measurement). Describe comparability of assessment methods if there is more than one group | 4-5 | The examinations were carried out using the diagnostic and rehabilitation device Luna EMG (EGZOTech Sp.z oo Gliwice, Poland). The assessments were carried out by two independent raters, under the same conditions and during the same time period. Two examinations of proprioception in relation to upper limb function were performed two weeks apart.  Luna EMG is a diagnostic and rehabilitation robotic device and its operation is based on reactive electromyography designed to train the sensorimotor cortex. The bioelectrical signals (EMG) obtained from the patient’s muscles show that the movement is active (Rinderknecht et al. 2018, Oleksy et. al., 2022).  Before the start of the examination the Luna EMG device was calibrated, in order to avoid errors in the measurements. The assessments were performed in line with the methodology provided by the manufacturer and in compliance with the scientific reports contained in the evaluation of the device in healthy subjects (Leszczak et al., 2024). During the measurements, the patients remained in a sitting position, with the upper limb from the shoulder joint aligned with the trunk, and extended in the elbow, hips flexed at 90°. The trunk and the assessed limb were stabilised using straps. Each measurement comprised four tests performed in succession in each upper extremity. The initial elbow joint position was 0° and the target position of elbow flexion was 60°; the movement was performed either actively or passively (Leszczak et al., 2024) (Figure 2).  The elbow was passively moved to the defined target position, to enable active assessment of the joint position. It was maintained in the same position for five seconds, so that the patient would remember the specific alignment. Then it was passively moved back to the initial position, which was then maintained for three seconds. After that the patient actively moved the limb in order to return to the target position (Oleksy et al. 2022; Neurorehabilitation robots. 2023; Leszczak et al., 2024). The device recorded the position once the patient verbally reported that they believed the elbow was back in the target position. |
| Bias | 9 | Describe any efforts to address potential sources of bias | 4-5 | The examinations were carried out using the diagnostic and rehabilitation device Luna EMG (EGZOTech Sp.z oo Gliwice, Poland). The assessments were carried out by two independent raters, under the same conditions and during the same time period. Two examinations of proprioception in relation to upper limb function were performed two weeks apart.  Luna EMG is a diagnostic and rehabilitation robotic device and its operation is based on reactive electromyography designed to train the sensorimotor cortex. The bioelectrical signals (EMG) obtained from the patient’s muscles show that the movement is active (Rinderknecht et al. 2018, Oleksy et. al., 2022).  Before the start of the examination the Luna EMG device was calibrated, in order to avoid errors in the measurements. The assessments were performed in line with the methodology provided by the manufacturer and in compliance with the scientific reports contained in the evaluation of the device in healthy subjects (Leszczak et al., 2024). During the measurements, the patients remained in a sitting position, with the upper limb from the shoulder joint aligned with the trunk, and extended in the elbow, hips flexed at 90°. The trunk and the assessed limb were stabilised using straps. Each measurement comprised four tests performed in succession in each upper extremity. The initial elbow joint position was 0° and the target position of elbow flexion was 60°; the movement was performed either actively or passively (Leszczak et al., 2024) (Figure 2).  The elbow was passively moved to the defined target position, to enable active assessment of the joint position. It was maintained in the same position for five seconds, so that the patient would remember the specific alignment. Then it was passively moved back to the initial position, which was then maintained for three seconds. After that the patient actively moved the limb in order to return to the target position (Oleksy et al. 2022; Neurorehabilitation robots. 2023; Leszczak et al., 2024). The device recorded the position once the patient verbally reported that they believed the elbow was back in the target position. |
| Study size | 10 | Explain how the study size was arrived at | 3 | The minimum sample size was determined prior to the study, using a sample size calculator. The procedure identified a population size of 71 patients with chronic stroke. Ultimately, 126 patients with chronic stroke were enrolled for the study. |

Continued on next page

| Quantitative variables | 11 | Explain how quantitative variables were handled in the analyses. If applicable, describe which groupings were chosen and why | N/A |  |
| --- | --- | --- | --- | --- |
| Statistical methods | 12 | (*a*) Describe all statistical methods, including those used to control for confounding | 5 | The mean and standard deviation values were first calculated for each series of measurements, and then for the differences between the series of measurements compared in the study. The significance of differences in the average level of the two measurement series was assessed using the t-test for dependent samples in which no significant values should be observed, but it should be noted that this is not a key factor in assessing the agreement of measurements. A comparative analysis of the measurement series was performed using Pearson's linear correlation coefficient, as well as the key measure of agreement between two measurements, i.e., intraclass correlation coefficient (ICC). Bland-Altman method was applied as an alternative measure of agreement.  The value of p<0.05 was assumed to reflect statistical significance. |
|  |  | (*b*) Describe any methods used to examine subgroups and interactions | 5 | The significance of differences in the average level of the two measurement series was assessed using the t-test for dependent samples in which no significant values should be observed, but it should be noted that this is not a key factor in assessing the agreement of measurements. A comparative analysis of the measurement series was performed using Pearson's linear correlation coefficient, as well as the key measure of agreement between two measurements, i.e., intraclass correlation coefficient (ICC). Bland-Altman method was applied as an alternative measure of agreement. |
|  |  | (*c*) Explain how missing data were addressed | 4 | Regular repetitions were carried out on a group of 126 people, among whom there are no missing data. |
|  |  | (*d*) *Cohort study*—If applicable, explain how loss to follow-up was addressed  *Case-control study*—If applicable, explain how matching of cases and controls was addressed  *Cross-sectional study*—If applicable, describe analytical methods taking account of sampling strategy |  | The study was not a cohort study. |
|  |  | (*e*) Describe any sensitivity analyses |  | N/A |
| Results | | | | |
| Participants | 13* | (a) Report numbers of individuals at each stage of study—eg numbers potentially eligible, examined for eligibility, confirmed eligible, included in the study, completing follow-up, and analysed | 4 | There were 200 patients with stroke in the rehabilitation and spa therapy hospital at the time of the study. Based on the inclusion and exclusion criteria, 147 patients were initially qualified to participate, however after medical examination eight individuals were excluded. Ultimately 130 patients took part in Examination I, and 126 patients participated in Examination II (Figure 1). |
|  |  | (b) Give reasons for non-participation at each stage | 4 | There were 200 patients with stroke in the rehabilitation and spa therapy hospital at the time of the study. Based on the inclusion and exclusion criteria, 147 patients were initially qualified to participate, however after medical examination eight individuals were excluded. Ultimately 130 patients took part in Examination I, and 126 patients participated in Examination II (Figure 1). |
|  |  | (c) Consider use of a flow diagram | 4 | Figure 1. Flow diagram. |
| Descriptive data | 14* | (a) Give characteristics of study participants (eg demographic, clinical, social) and information on exposures and potential confounders | 4 | The study group comprised 126 individuals, including 78 women (61.9%) and 48 men (38.1%) (Table 1).  The youngest participant was aged 45 (Min. 45.0), and the oldest 75 years (Max. 75.0), the mean age being nearly 60 years ( = 59.9). The respective mean values of body weight (in kilograms), body height (in centimetres) and body mass index (BMI) were 68.3kg ( = 68.3), 170cm ( = 170.0) and 23.6kg/m2 |
|  |  | (b) Indicate number of participants with missing data for each variable of interest | 4 | There were 200 patients with stroke in the rehabilitation and spa therapy hospital at the time of the study. Based on the inclusion and exclusion criteria, 147 patients were initially qualified to participate, however after medical examination eight individuals were excluded. Ultimately 130 patients took part in Examination I, and 126 patients participated in Examination II (Figure 1). |
|  |  | (c) *Cohort study*—Summarise follow-up time (eg, average and total amount) |  | N/A |
| Outcome data | 15* | *Cohort study*—Report numbers of outcome events or summary measures over time |  | N/A |
|  |  | *Case-control study—*Report numbers in each exposure category, or summary measures of exposure |  | N/A |
|  |  | *Cross-sectional study—*Report numbers of outcome events or summary measures |  | N/A |
| Main results | 16 | (*a*) Give unadjusted estimates and, if applicable, confounder-adjusted estimates and their precision (eg, 95% confidence interval). Make clear which confounders were adjusted for and why they were included |  | Do not include any measurement errors. Due to the diversity of the study group and the repetition of measurements, there was no such need. |
|  |  | (*b*) Report category boundaries when continuous variables were categorized |  | N/A |
|  |  | (*c*) If relevant, consider translating estimates of relative risk into absolute risk for a meaningful time period |  | N/A |

Continued on next page

| Other analyses | 17 | Report other analyses done—eg analyses of subgroups and interactions, and sensitivity analyses |  | N/A |
| --- | --- | --- | --- | --- |
| Discussion | | | | |
| Key results | 18 | Summarise key results with reference to study objectives | 7-9 | The research has shown that Luna EMG is a reliable tool in the evaluation of upper limb proprioception. Measurements made with Luna EMG show high inter-rater and intra-rater reliability of the assessment of the proprioceptive sensation of the upper limb in individuals with stroke.  In the international literature there are no scientific reports investigating this subject matter, i.e. Luna EMG aided assessment of upper limb proprioception in patients with stroke. The only related studies published so far have presented evidence showing reliability of this tool in healthy populations, when the device was applied to assess the sense of knee position (Oleksy et al., 2022) and to measure upper limb proprioception (Leszczak et al., 2024). The former study showed high reliability (ICC=0.866–0.982) of Luna EMG aided assessments of both knee flexion and extension in active and passive modes in healthy individuals (Oleksy et al., 2022). Likewise, the latter study demonstrated a high consistency (ICC=0.969-0.997) of upper limb proprioception measurements performed using the device (Leszczak et al., 2024).  Another reason for undertaking this research lies in the fact that very few studies so far have investigated upper limb proprioception in patients with stroke (Rand 2018; Kiper et al., 2015; Ocal et al., 2020), however the findings reported are rather promising. As an example, Kiper et al. demonstrated that proprioceptive training may lead to improvements in patients with upper limb paralysis after subacute stroke (Kiper et al., 2015). Furthermore, a study by Ocal et al. showed that, compared to a conventional therapy, proprioceptive training of the upper limb more effectively increases the frequency and quality of movements performed with the upper limb by patients at a chronic stage post-stroke (Ocal et al., 2020)]. Therefore, we believe that it is necessary to continue research focusing on various issues associated with upper limb proprioception post-stroke. Our findings show that the Luna EMG multifunction robotic device is a reliable tool in the evaluation of upper limb proprioception in patients with chronic stroke. It is a well-established fact that diagnostic assessments are essential for proper planning and monitoring of training-based therapies (Sarzyńska-Dlugosz 2023), therefore, in further studies we intend to evaluate the effectiveness of Luna EMG in assessing the effects of upper limb proprioceptive training in patients after stroke.  In summary of these considerations we can say that this study presents the first scientific evidence related to evaluation of upper limb proprioception in patients with chronic stroke, performed using the Luna EMG multifunction robotic device, and the findings are consistent with results of other studies involving healthy participants, and they demonstrate high reliability of the tool in evaluating upper limb proprioception in patients at a chronic phase after stroke. |
| Limitations | 19 | Discuss limitations of the study, taking into account sources of potential bias or imprecision. Discuss both direction and magnitude of any potential bias | 9 | The study has some limitations, most importantly, evaluation of upper limb proprioception performed with the Luna EMG diagnostic and rehabilitation device was not supported with results acquired using proprioception assessment scales and tests. However, we are planning further research in which effects of stroke rehabilitation will be evaluated using Luna EMG as well as assessment scales and tests. |
| Interpretation | 20 | Give a cautious overall interpretation of results considering objectives, limitations, multiplicity of analyses, results from similar studies, and other relevant evidence | 9 | The study demonstrates that the Luna EMG multifunction robotic device is a reliable tool in the evaluation of upper limb proprioception. Measurements made with Luna EMG show high inter-rater and intra-rater agreement in the assessment of the proprioceptive sensation of the upper limb in patients with stroke. |
| Generalisability | 21 | Discuss the generalisability (external validity) of the study results | 9 | The study demonstrates that the Luna EMG multifunction robotic device is a reliable tool in the evaluation of upper limb proprioception. Measurements made with Luna EMG show high inter-rater and intra-rater agreement in the assessment of the proprioceptive sensation of the upper limb in patients with stroke. |
| Other information | |  | | |
| Funding | 22 | Give the source of funding and the role of the funders for the present study and, if applicable, for the original study on which the present article is based | 9 | The research presented in this paper was co-financed by the European Union from the European Regional Development Fund, Smart Growth Operational Programme, grant no. POIR.01.01.01-00-2077/15 ”Development of innovative methods of automatic diagnostics and rehabilitation using robots and bioelectric measurements” |

*Give information separately for cases and controls in case-control studies and, if applicable, for exposed and unexposed groups in cohort and cross-sectional studies.

**Note:** An Explanation and Elaboration article discusses each checklist item and gives methodological background and published examples of transparent reporting. The STROBE checklist is best used in conjunction with this article (freely available on the Web sites of PLoS Medicine at http://www.plosmedicine.org/, Annals of Internal Medicine at http://www.annals.org/, and Epidemiology at http://www.epidem.com/). Information on the STROBE Initiative is available at www.strobe-statement.org.
